# Central command suite: futureproofing next-generation surgical environments to embrace the digital operating room

**DOI:** 10.1007/s11548-024-03278-w

**Published:** 2024-12-21

**Authors:** Carlos L. Amato, Chengyuan Yang, Niloofar Badihi, Lukas Bernhard, Osman Ratib, Barbara Seeliger, Dirk Wilhelm

**Affiliations:** 1 Cannon Design, Architecture and Planning, Los Angeles, CA USA; 2 Research Group MITI, TUM University Hospital, School of Medicine and Health, Technical University of Munich, Munich, Germany; 3 Agora Care SA, Plan-les-Ouates, Switzerland; 4 Institute of Image-Guided Surgery, IHU Strasbourg, Strasbourg, France; 5 Department of Digestive and Endocrine Surgery, University Hospitals of Strasbourg, Strasbourg, France; 6 ICube, UMR7357, CNRS, INSERM U1328 RODIN, Université de Strasbourg, Strasbourg, France; 7 IRCAD, Research Institute Against Digestive Cancer, Strasbourg, France; 8 Department of Surgery, TUM University Hospital, School of Medicine and Health, Technical University of Munich, Munich, Germany

**Keywords:** Multidisciplinary collaboration, Central command suite, Surgery intelligence, Surgical communications, Surgery innovation

## Purpose

Multidisciplinary board meetings have been proven advantageous in medical decision-making and have become the standard in oncology. In areas with time-critical decision-making, a control room serves the same purpose and is continuously staffed with all expertise required.

There is a misconception suggesting that if team members are dealing with data alone, there is no need to physically bring people together as they could communicate entirely via IT networks [1]. Our research indicates that to integrate the use of advanced and artificial intelligence, the surgical platform of the future requires a central information hub to enable and enhance transformative multidisciplinary collaboration and teamwork [2].

The space of the central command suite (CCS) is designed to plan surgical workflows and to control their execution using model-guided medicine (MGM). It is a technologically advanced environment that supports effective decision-making, efficient operations management, and overall enhanced patient care. The CCS serves as the central control hub to organize and optimize perioperative data and is thus interconnected to the entire infrastructure that is relevant throughout a surgical patient journey, in addition to the core OR and imaging facilities. Its main objective is to facilitate direct, in-person connections between surgeons, interventionalists, radiologists, other clinicians, scientists, and stakeholders to amplify communication and coordinate key decisions.

To ensure CCS effectiveness and efficiency, its design should consider several key factors, including collaboration and workflow support, ergonomics and functionality, scalability and flexibility, integration of all communication and information systems, data analytics and visualization, redundancy and backup systems, security, privacy, accessibility, compliance, education, research, and training [3].

The CCS covers different time and process scales, ranging from preoperative planning to postoperative observation and from a single intervention to the orchestration of an OR suite with hundreds of OR theaters. It is the central connection behind interventional medicine to orchestrate all interventions including the required resources, to observe the execution including all related processes, and to adjust as necessary (e.g., troubleshooting) [4].

With this short communication paper, we present a new concept for creating a place that brings together all the intelligence required in a future digital operating room (DOR) platform. The most relevant and promising concepts are identified, and the spatial/procedural requirements for integrating such systems into the DOR environment are discussed in their essence.

## Methods

The CCS concepts presented in this abstract result from internal, multidisciplinary discussions and experiences from current hospital projects and on concepts for the healthcare of tomorrow, including the Think Tank on the “Digital Operating Room (OR 2040) and Model-Guided Medicine” kick-off sessions in Rothaus, Black Forest, Germany, in October 2022 and 2023, Hospital of the Future project, and operating room of the future (CARS 2022), complemented by further dedicated literature research.

Our study explicitly focuses on promoting in-person interaction and collaboration between specialists performing a variety of surgical and imaging functions that are key to patient outcomes [5]. Inspiration comes from the NASA mission control which is divided into different teams which all do contribute with their expertise on a main mission—or in our example to run the DOR. Likewise, concepts are to be found in ships/large cruisers, fire and police departments, and at the stock exchange, where ad-hoc decisions have to be made on the basis of high-volume data and comprehensive knowledge. Our vision is strongly related to the concept of MGM which not only applies the idea of a digital patient twin, but which replicates any agent and process related to the treatment of a patient in a digital model, which are fed by online data, allowing not only for the automatization of processes, but also for their planning, optimization, and control throughout the treatment process [6]. MGM can be understood as a comprehensive digital twin, which, however, is firmly linked to the real processes, so that digital actions will have an effect in the real world (haptic internet). As healthcare processes are roughly unpredictable in nature, and acute situations (complications, emergencies, lacks in supply chain, etc.) ask for dynamic adoption and continuous revision of therapeutic pathways, a control center to design, manage, and control such activities becomes necessary. The present concept is focused on the OR/Interventional theater, in contrast to a command center to run the entire hospital.

## Results

### General requirements

The key functions required in the CCS include:

1. Pre-procedure planning.
2. Procedure simulation, patient modelling  (“Digital Twin” creation, 3D anatomic modeling unit, 3D printing) and procedure warmup.
3. Creation and coordination of advanced imaging (image overlay, enhanced reality, etc.) [7].
4. Post-procedure analysis
5. Research and education
6. Robotics engineering
7. IT infrastructure coordination (remote monitoring (clinical), ambient intelligence, AI answers engine)
8. Communication and operations (access to PACS/EMR, intra- and extra-department, patient flow, resource utilization, incoming admissions)
9. Predictive resource delivery systems (predictive space utilization (PACU bypass, etc.) digital assistant/navigator, automatic and predictive room setup for each specific surgery case)
10. Staff management (integration of staff shortage info and best spatial distribution to ensure OR turnaround)
11. Efficiency management (patient throughput, financial resources, cost-effectiveness of decisions, and schedules) [8].
12. Operation of preplanned processes including management of unintended events and complications
13. Further dedicated studies for research and education purposes [9].

In addition, the spatial implications for each of these functions were categorized into three groups:

High Changes departmental spatial relationships and/or has structural implications
Medium Changes room spatial relationships
Low Changes room requirements within the boundaries of a room typology

As the CCS represents an area where functionality and architecture/design are connected interdependently more than ever, both fields are described more in detail.

### Functional design concept

#### Preparation

The concept of MGM is based on the availability and the access to data of any kind (imaging, patient data, resources, staff, etc.) and temporal relation (pre-, peri-, postoperative, patient reports and history, PROMS, etc.), so the digital interconnection to any of such resources is a core prerequisite. In future, ideally a team of people (medical, technical, administrative, etc.) will be responsible for the design, management and orchestration of such models and will work in close proximity to one another in defined areas of the CCS. Here, they will prepare the different components relevant to a treatment process on the patient level. Different patient-specific treatment models will be merged for the construction of the OR planning and will all of them define the required resources necessary for their execution (staff, devices, room functionalities, duration, pre- and postoperative monitoring and care, etc.). The responsibility for the optimized scheduling of interventions is given to another team of experts, which needs to be closely connected to the resource managing team. The preparation part of the CCS designs OR schedules weeks before their execution but revises them on a daily basis depending on their executability and effectiveness.

#### Execution

**Fig. 1:**
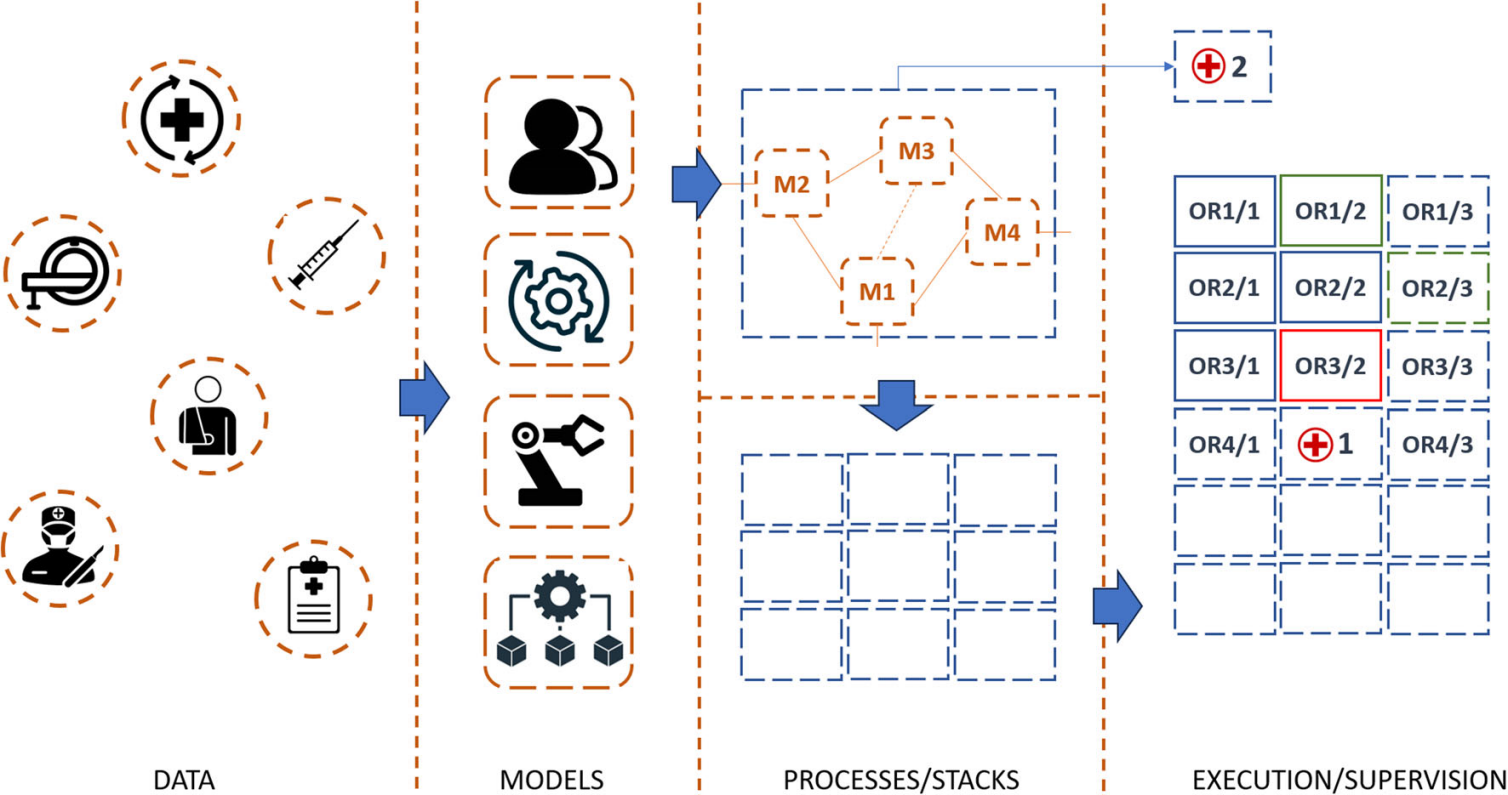
In MGM and in a semi-autonomous approach data will be processed and integrated into models on which basis treatment pathways will be formulized. Stacking of such pathways will facilitate optimized OR schedules, which serve as a planning, but which need to be supervised for complications and in case of emergencies. (Modified concept according to H.U. Lemke [1])

The second core functionality within the CCS, and the more demanding one, is the observation of running processes and the real-time adaptation (e.g., troubleshooting) [10]. Although any adoption and necessary modification of the preplanned schedule will impact on the preparation of subsequent interventions and resource management, decisions to be made here are much more time-critical. Accordingly, the executive team needs to be closely connected to the planning and acts as a superior authority and at the highest functional level within the CCS. The coordinating authority must be directly supported by a responsible party from the planning and design level, so that necessary modifications can be directly dispatched to the teams in charge (Fig. 1).

### Room concept

The architectural design of the CCS follows the functional requirements and therefore is organized in different teams and executive levels. The core area is provided to the executive coordinators and their teams which will have supervision to all running processes, and which adapt the planned schedule in case of complications (exceeding duration, missing resource, modification of surgery) or emergencies coming in. Large video screens inform the coordinators on the current state of tasks execution and allow for adaptive zooming to different granularity levels, either to observe the overall processes or to identify interfering issues [11]. In the same room the planning unit merges patient pathways and surgical process models for a perfect OR schedule, but serves also responsible for the preparation of workflows for acute cases and emergencies. This core area is surrounded by the many modeling and data processing units, which are obligated with the design and patient and process-individual adaptation of medical models. Although many of these processes will be realized as automatized approaches and facilitated by AI, the curation of data, validation of models and final approval of processes must remain a human responsibility and follow ethical and legal considerations [12]. For such purposes the teams at the different units will have access to all necessary data, but will also be equipped with necessary technologies, e.g., AR/XR devices and simulation suites, segmentation and annotation tools, etc.

**Fig. 2:**
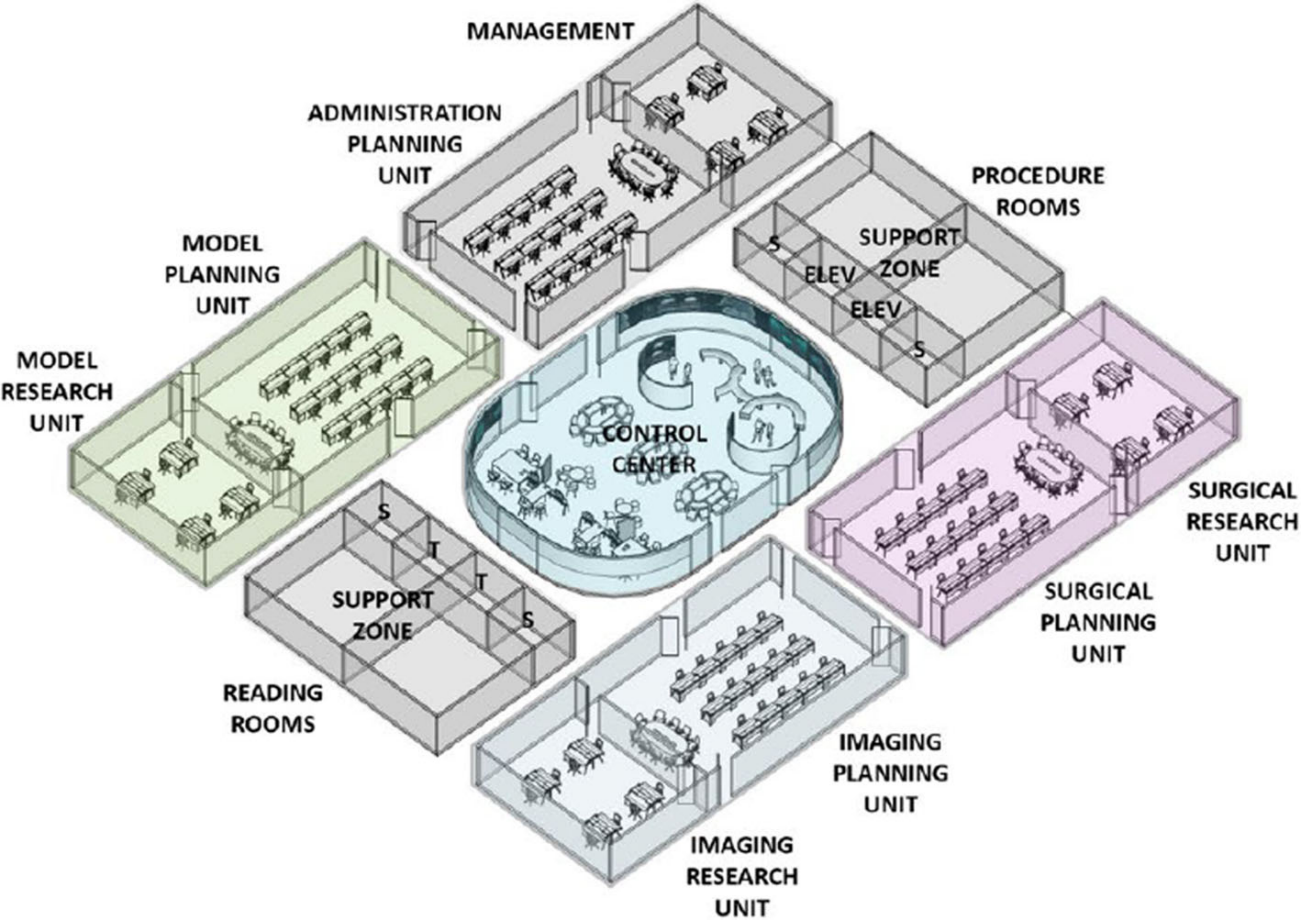
Modular design strategy with potential command suite functions

**Fig. 3:**
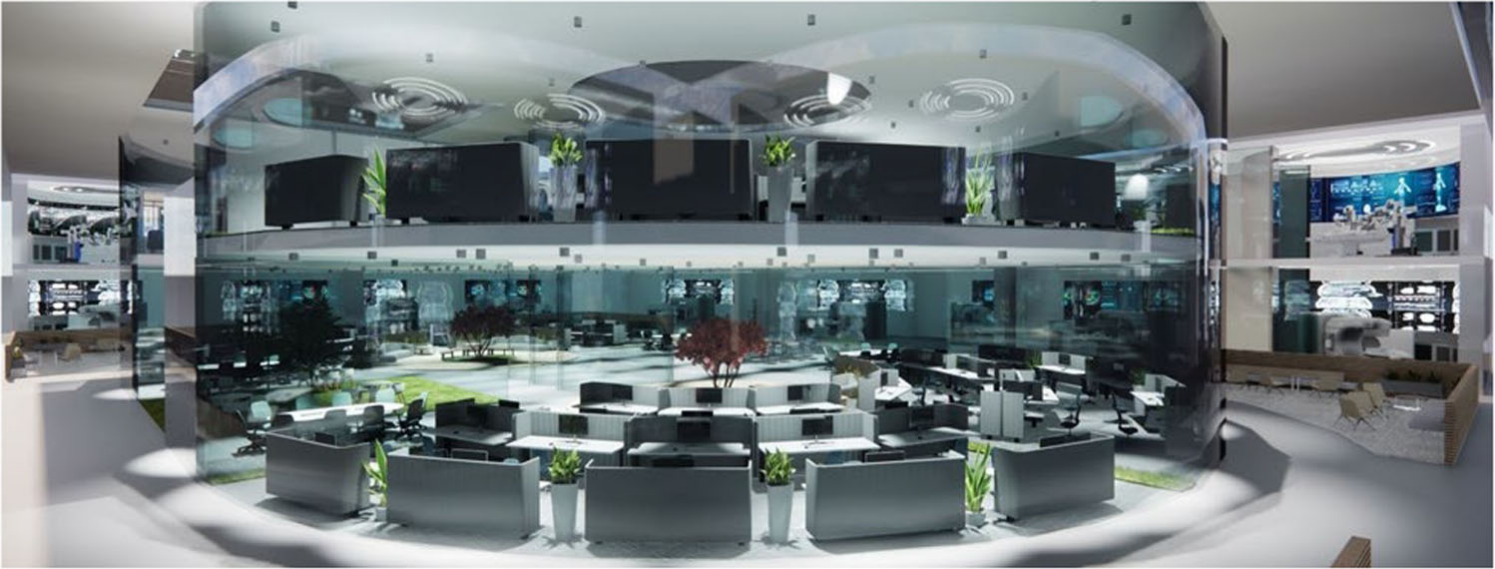
Command suite interior view (Architect rendering)

Figures 2 and 3 show a first conceptual visualization of a future central command suite as a workspace for human–digital collaboration. In our presentation, we will elaborate on such spatial considerations and discuss the potential of different concepts in more detail.

Our team determined several benefits associated with our CCS concept including:

*Centralized data management* Serves as a hub for collating and managing these data efficiently. It ensures that all relevant information is accessible in one place, leading to more informed decision-making and better coordination among medical staff [13].

*Enhanced communication and collaboration* Acts as a control center for coordinating tasks, sharing updates, and responding to emergencies promptly. This integrated approach can significantly improve the efficiency and effectiveness of surgical procedures [14].

*Integration with advanced technologies* Assists in tasks such as patient monitoring, surgical planning, and postoperative care, enhancing patient outcomes and surgical precision.

*Scalability for future innovations* Offers a scalable platform that can adapt to future advancements. Whether it’s the integration of new AI algorithms, telemedicine capabilities, or advanced diagnostic tools, having a dedicated space that is designed to evolve with technology ensures that the surgery department remains at the forefront of medical innovation.

*Improved patient outcomes* Contributes to more accurate diagnoses, more precise surgeries, and better overall patient care. By leveraging AI and data analytics, the suite can support early identification of potential complications, thus improving the rate of uncomplicated surgical procedures.

*Operational efficiency and cost-effectiveness* By centralizing operations and utilizing advanced computer science including AI for data analysis and decision-making, a central command suite can streamline workflows, reduce redundancy, and optimize resource utilization. This efficiency not only saves time but also can result in significant cost savings for the medical center [15].

Our presentation describes the process to understand and decide how to implement each function early in the design and planning process to ensure the resulting concept can accommodate the vision.

## Conclusion

Ultimately, the goal of any healthcare facility is to improve patient care. A central command suite for a surgery department aligns perfectly with the needs of future healthcare facilities, particularly in managing complex data and integrating future digital and AI advancements. It not only enhances operational efficiency and patient care but also ensures that a future DOR in a surgery department is well-prepared to embrace future technological innovations.

## References

[1] Shortliffe EH, Chiang MF (2021) Biomedical data: their acquisition, storage, and use. 10.1007/978-3-030-58721-5_2

[2] Li J, Robertson T, Hansen S, Mansfield T, Kjeldskov J (2008) Multidisciplinary medical team meetings: a field study of collaboration in health care. 10.1145/1517744.1517766

[3] Sokolov S (2023) Design of decision support systems with neural networks in biomedical data analysis. In: 2023 4th international conference on communications, information, electronic and energy systems (CIEES), Plovdiv, Bulgaria, 2023, pp 1–7. 10.1109/CIEES58940.2023.10378726

[4] Overdyk FJ, Dowling O, Newman S, Glatt D, Chester M, Armellino D, Cole B, Landis GS, Schoenfeld D, DiCapua JF (2016) Remote video auditing with real-time feedback in an academic surgical suite improves safety and efficiency metrics: a cluster randomised study. BMJ Quality Safety 25(12):947–953. 10.1136/bmjqs-2015-004226 26658775 10.1136/bmjqs-2015-004226 PMC5256234

[5] Krauss O, Angermaier M, Helm E (2016) Multidisciplinary team meetings—a literature based process analysis. In: Renda M, Bursa M, Holzinger A, Khuri S (eds) Information technology in bio- and medical informatics. ITBAM 2016. lecture notes in computer science, vol 9832. Springer, Cham. 10.1007/978-3-319-43949-5_8

[6] Lennerz JK, Salgado R, Kim GE, Sirintrapun SJ, Thierauf JC, Singh A, Indave I, Bard A, Weissinger SE, Heher YK, de Baca ME, Cree IA, Bennett S, Carobene A, Ozben T, Ritterhouse LL (2023) Diagnostic quality model (DQM): an integrated framework for the assessment of diagnostic quality when using AI/ML. Clinical Chemistry and Laboratory Medicine (CCLM), vol 61, no 4, pp 544–557. 10.1515/cclm-2022-1151 10.1515/cclm-2022-1151 36696602

[7] Nakanoko T, Oki E, Ota M et al (2023) Real-time telementoring with 3D drawing annotation in robotic surgery. Surg Endosc 37:9676–9683. 10.1007/s00464-023-10521-z 37935920 10.1007/s00464-023-10521-z

[8] Tomashev R, Alshiek J, Shobeiri SA (2023) Improving the operating room efficiency through communication and lean principles. In: Chilingerian JA, Shobeiri SA, Talamini MA (eds) The new science of medicine and management. Springer, Cham. 10.1007/978-3-031-26510-5_4

[9] Mirkin B (2013) Methods for interpretation of data in medical informatics. In: Kountchev R, Iantovics B (eds) Advances in intelligent analysis of medical data and decision support systems. Studies in computational intelligence, vol 473. Springer, Heidelberg. 10.1007/978-3-319-00029-9_2

[10] Charalambous A, et al (2021) Developing and utilizing digital technology in healthcare for assessment and monitoring. 10.1007/978-3-030-60697-8

[11] Cheikh Youssef S, Haram K, Noël J et al (2023) Evolution of the digital operating room: the place of video technology in surgery. Langenbecks Arch Surg 408:95. 10.1007/s00423-023-02830-7 36807211 10.1007/s00423-023-02830-7 PMC9939374

[12] Perc M, Hojnik J (2022) Social and legal considerations for artificial intelligence in medicine. In: Lidströmer N, Ashrafian H (eds) Artificial intelligence in medicine. Springer, Cham. 10.1007/978-3-030-64573-1_266

[13] Perger P, Buccioli M, Agnoletti V, Padovani E, Gambale G (2014) Operating room efficiency improving through data management. In: Roa Romero L (eds) XIII mediterranean conference on medical and biological engineering and computing 2013. IFMBE Proceedings, vol 41. Springer, Cham. 10.1007/978-3-319-00846-2_324

[14] Robertson T, Li J, O’Hara K et al (2010) Collaboration within different settings: a study of co-located and distributed multidisciplinary medical team meetings. Comput Supported Coop Work 19:483–513. 10.1007/s10606-010-9124-9

[15] Ferreira DC, Nunes AM, Marques RC (2020) Operational efficiency vs clinical safety, care appropriateness, timeliness, and access to health care. J Prod Anal 53:355–375. 10.1007/s11123-020-00578-6

